# Large atypical parathyroid tumor - a diagnostic conundrum

**DOI:** 10.4322/acr.2024.514

**Published:** 2024-09-12

**Authors:** Saikat Mitra, Shouvanik Satpathy, Devmalya Banerjee, Sugat Sanyal

**Affiliations:** 1 Peerless Hospital and B.K. Roy Research Center, Department of Histopathology and Lab services, Kolkata, West Bengal, India; 2 Peerless Hospital and B.K. Roy Research Center, Department of ENT and Head Neck Surgery, Kolkata, West Bengal, India

**Keywords:** Parathyroid adenoma, Aspiration, fine Needle, hyperparathyroidism, Histopathology, Parathyroid Carcinoma

## Abstract

Atypical parathyroid tumor (APT) is a rare neoplasm of the parathyroid gland, which shows atypical cytological or architectural features and lacks definite diagnosis criteria for malignancy. These cases can cause diagnostic challenges owing to their rarity and similarity with thyroid neoplasm on imaging and fine needle aspiration cytology. Also, differentiating APT from giant parathyroid adenoma or parathyroid carcinoma can be challenging based on clinical, imaging or cytological features. A 49-year-old male presented with clinical features of hyperparathyroidism. On laboratory evaluation, his serum calcium and serum parathyroid hormone was elevated. Imaging studies suggested a possibility of left inferior parathyroid neoplasm, and fine needle aspiration cytology showed features suggestive of parathyroid neoplasm. However, exact categorization of parathyroid tumor was difficult in pre-operative work-up. Possibilities of giant parathyroid adenoma as well as parathyroid carcinoma were considered. A final diagnosis of an atypical parathyroid tumor was made after detailed histopathological evaluation given focal capsular invasion but lack of unequivocal evidence of malignancy in the resected specimen. APT is a rare neoplasm of uncertain malignant potential. Knowledge of the radiological and pathological features will be helpful in accurately identifying the lesion and avoiding misdiagnosis.

## INTRODUCTION

An atypical parathyroid tumor (APT) is a parathyroid neoplasm with atypical cytological and/or architectural features but lacks unequivocal capsular, vascular, perineural adjacent structures invasion or metastases.^1^ This tumor was previously known as Atypical Parathyroid Adenoma (APA); however, in the recent update of WHO 5^th^ edition of the endocrine tumor, the term APT is used.^2^ APTs usually occur sporadically, although familial and syndromic association is described, mainly in MEN1 syndrome.^3^ Patients may be asymptomatic or have features of hyperparathyroidism. Clinically, differentiating APT from parathyroid carcinoma can be difficult. Preoperative localization by imaging, including ultrasound, magnetic resonance imaging (MRI), and scintigraphy, can aid the diagnosis. However, large APTs adhered to thyroid or intrathyroidal parathyroid may challenge the differential with thyroid tumors. Atypical parathyroid tumor cytological features are non-specific and show morphological resemblance with thyroid neoplasm.^4^ Distinguishing atypical parathyroid tumors from carcinoma on cytology is not possible. Hence, solid clinicopathological and imaging correlation is mandatory. In this report, we describe a case of a middle-aged male patient in whom a diagnosis of APT was established after a thorough examination of the resected specimen with immunohistochemical evaluation. The diagnostic difficulties of APT in cytology and histopathology, along with its differentials, are discussed here.

## CASE REPORT

A 49-year-old male presented with fatigue, body aches, increased thirst, constipation, and increased frequency of urination. On the laboratory workup, his serum calcium level was (15.94 mg/dl (normal-8.5-10.2 mg/dl), phosphate level 2.2 mg/dl (normal-2.5-4.5 mg/dl), creatinine 0.91 mg/dl (normal 0.7-1.3 mg/dl) and estimated glomerular filtration rate was 103 ml/min/1.73m^2^ (normal: more than 90 ml/min/1.73m^2^). Imaging studies showed renal calculi. Systemic clinical evaluation was normal. The serum intact parathyroid hormone (iPTH) was (562 pg/ml, normal 14-72 pg/ml). A working diagnosis of primary hyperparathyroidism was made, and ultrasonography (USG) evaluation of the neck showed a hypoechoic well-defined mass of 35x25 mm was identified close to the thyroid left lobe, slightly left to the midline. The lesion was highly considered suspicious for malignancy based on the large size and hypoechogenicity.

**Figure 1:**
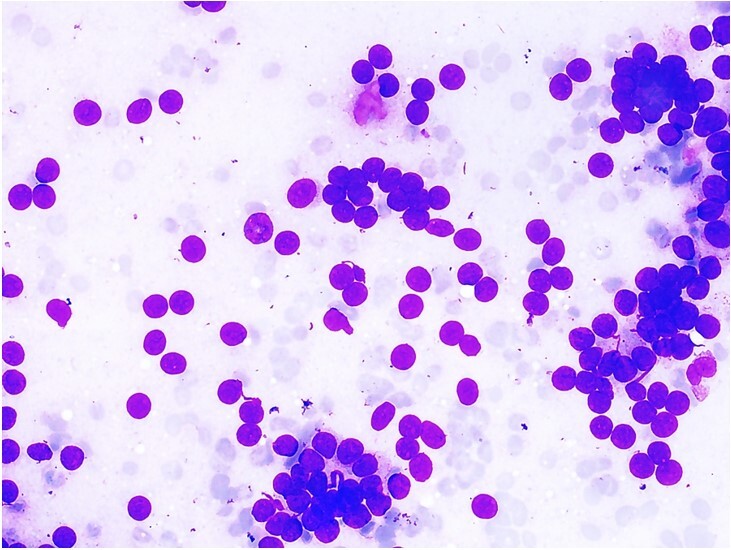
Fine needle aspiration smear of lesion shows cellular tumor arranged in clusters forming pseudoacini and singly scattered cells with mild nuclear atypia over a hemorrhagic background devoid of colloid (May-Grünwald-Giemsa, 100x).

USG-guided FNA showed monomorphic tumor cells arranged in singly scattered bare nuclei and loose clusters and pseudoacini were formed over a hemorrhagic background (Figure 1). No colloid was noted. The tumor cells had round nuclei, dense chromatin, and inconspicuous nucleoli, and scant to moderate cytoplasm. Features of malignancy in the form of marked pleomorphism, necrosis, and frequent mitosis were not identified in the cytology smears.

**Figure 2:**
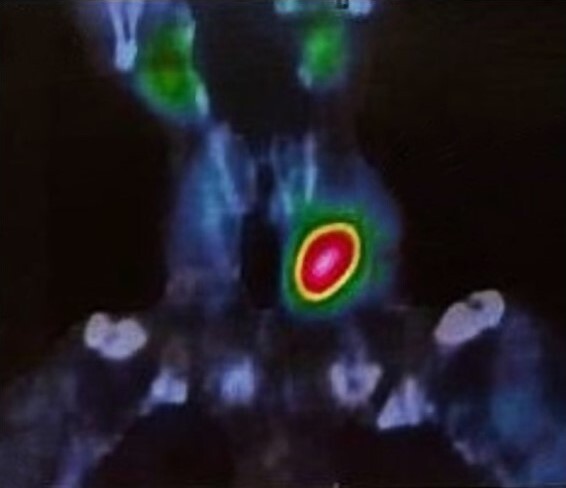
T-99m SPECT-CT shows increased tracer uptake in left lower lobe of thyroid.

The patient also underwent a Technetium-99m labeled single photon emission computed tomography (T-99m SPECT/CT) of the neck, highlighting intense tracer uptake in the left lower thyroid lobe (Figure 2).

**Figure 3:**
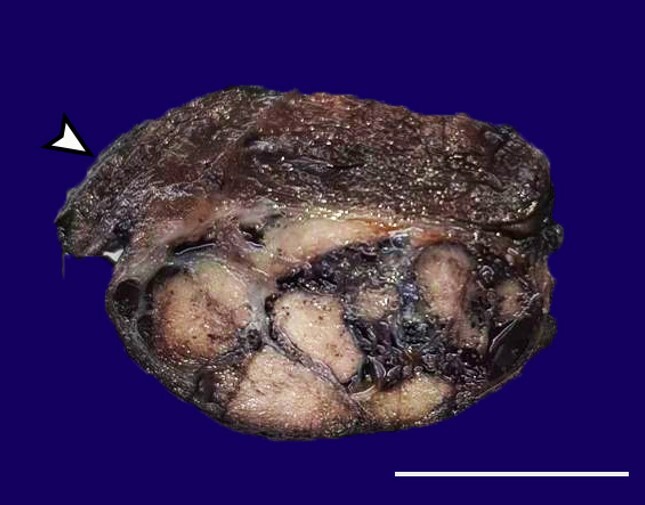
Macroscopic examination of the surgical specimen shows a capsulated, multinodular solid tumor closely adhered to the left lobe of the thyroid (arrowhead) (scale bar = 5 cm).

The preoperative diagnosis was left inferior parathyroid adenoma. During intraoperative evaluation, the tumor was seen to adhere to the dorsal surface of the left thyroid lobe. Several hemorrhagic left cervical lymph nodes were also identified. A malignant neoplasm was suspected, and the tumor was removed en-mass with the thyroid lobe. The right-sided parathyroid glands were identified and preserved. Selective left-sided neck dissection was also performed. The serum iPTH level was reduced to 202.8 pg/ml after the removal of the parathyroid tumor. On gross examination, a large, predominantly solid, encapsulated mass was identified as closely adhered to the posterior surface of the left thyroid lobe (Figure 3).

**Figure 4:**
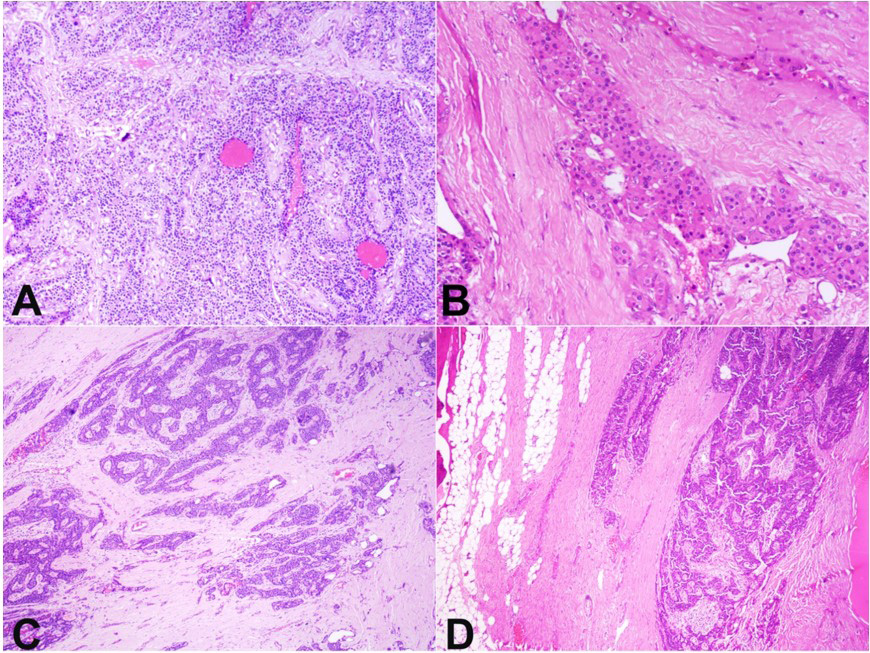
Photomicrographs of the tumor. **A –** Monomorphic tumor cells arranged in cords, trabeculae, and organoid pattern (H&E, 100x), **B –** Focal oncocytic changes in tumor cells (right side, H&E, 400x); **C –** Expanded and fibrotic capsule with extensive capsular infiltration by tumor cells (H&E, 100x); **D –** Tumor limited to capsule without any extracapsular spread (H&E, 100x).

The right lobe of the thyroid and the isthmus were normal. Microscopic evaluation showed a tumor arranged in sheets, trabeculae, and organoid nests. Focally, oncocytic cells were identified (Figure 4A and 4B).

The tumor cells showed minimal to mild nuclear atypia. Mitosis was infrequent (~1-2/10 HPF). Marked nuclear pleomorphism or necrosis were absent. The tumor showed diffuse capsular invasion and marked underlying stroma fibrosis. The capsule was markedly expanded (Figure 4C and 4D). No extracapsular extension was identified. All the resected lymph nodes were free of tumor.

**Figure 5:**
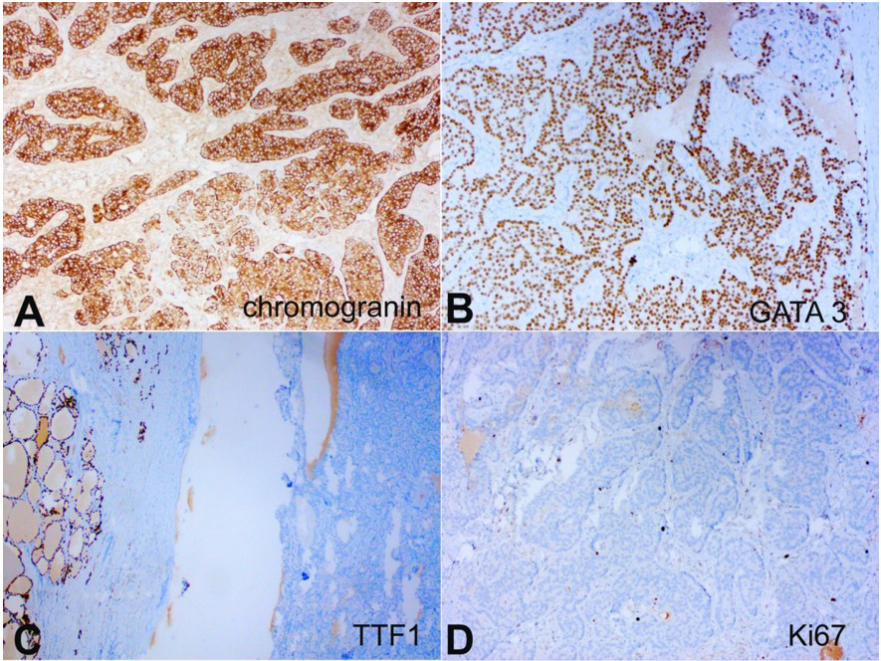
Photomicrographs of the tumor. **A –** Tumor cells show strong, diffuse cytoplasmic staining with Chromogranin A(100X); **B –** Tumor cells show strong, diffuse nuclear staining with GATA-3 (100X); **C –** Tumor cells are negative for TTF-1 (note positive staining in thyroid follicular epithelial cells in left) (100X); **D –** Ki-67 shows low proliferative index (100X).

On immunohistochemistry (IHC), the tumor cells were strongly and diffusely positive for Chromogranin A and GATA3 and were negative for TTF1, and Calcitonin. Ki67 labeling index was ~5% in tumor cells. IHC for Parafibromin and PTH were not performed due to the unavailability of the antibodies. (Figure 5A-D).

Given diffuse capsular invasion without any definite evidence of malignancy (lymphatic invasion, angioinvasion, perineural invasion, extracapsular extension to adjacent organ, histologically proven metastasis), a diagnosis of APT was rendered. The patient was kept on close follow-up.

## DISCUSSION

Atypical parathyroid tumor is a rare tumor of uncertain malignant potential.^5^ It was first included in the WHO classification of Endocrine tumors in 2004. The reported incidence of APT ranges from 0.5 to 4.4% in individuals undergoing surgery.^6^ Most cases occur sporadically, with a female preponderance. However, association with familial syndromes is also noted [e.g., Multiple Endocrine Neoplasia (MEN) 1 and MEN 2, single familial hyperparathyroidism (FIHP), hyperparathyroidism “jaw tumor” (HPTJT)].^6^ Loss of heterozygosity of RB1, CDC73, and CDKN2A genes have been associated with APT development.^7^ In the recent 5^th^ edition of WHO classification of parathyroid neoplasm, the term ‘atypical parathyroid adenoma’ has been replaced by ‘atypical parathyroid tumor’ to address the uncertain nature and the potential malignant transformation.^2^ The average size of APT is 30 mm (range 10-72 mm), and nearly half of them show adherence to adjacent organs, similar to the index case.^6^ Microscopically, these tumors show solid, trabecular, and monotonous sheet-like architecture; however, they lack the definite criteria for malignancy, which include(i) extracapsular spread to adjacent organs, (ii) lymphatic invasion,(iii) angioinvasion,(iv) perineural invasion,(v)histological confirmed metastasis.^2^ On histology, APTs are often difficult to differentiate from parathyroid adenoma (PA) or parathyroid carcinoma (PC). Large adenomas and those with prior fine needle aspiration history can show stroma fibrosis and focal capsular invasion.^8^ However, a diffuse capsular invasion should suggest the diagnosis of APT over PA. In our case, multifocal diffuse capsular invasion favored APT instead of sclerosed PA. Distinguishing PC from APT requires thorough sampling of the resected specimen to determine the above five criteria. APTs can be confused with medullary carcinoma of the thyroid because of the neuroendocrine nature of both tumors and close histological features. However, the presence of spindled to plasmacytoid cells and identification of amyloid in the stroma helps in distinguishing MTC from APT.^9^

IHC can help in distinguishing APT from its mimics. While MTC and APTs are positive for neuroendocrine markers, most MTCs are positive for TTF-1, while GATA-3 positivity confirms the parathyroid origin.^10^ Calcitonin is a helpful marker to distinguish the two, as calcitonin is expressed in MTC. Several IHC markers, including p27, CyclinD1, RB1, BCL2, MDM2, p53, galectin 3, and PGP 9.5, have been studied in parathyroid tumors; however, their use in routine clinical diagnostics is yet to be validated.^11,12^ The Ki67 labeling index of more than 5% has been associated with malignant behavior.^13^ In our case, the Ki67 labeling index was overall low, with only a few hotspots showing ~5% positivity. Absent nuclear parafibromin immunoreactivity indicates biallelic *CDC73* inactivation, commonly associated with germ-line mutation, which has shown utility in predicting poor outcome in APTs.^14^ Parafibromin immunohistochemistry could not be performed in our case due to the unavailability of the primary antibody.

The available literature on cytology diagnosis of APT is limited. However, it is well documented that parathyroid tumors are difficult to diagnose on cytology without proper clinical and imaging evaluation. Because of its proximity to the thyroid and overlapping cytomorphology, parathyroid tumors are often misdiagnosed as thyroid neoplasm.^15^ Few authors reported poor diagnostic sensitivities for FNAC (65% and 40.4%).^15,16^ The presence of follicular structures, oxyphilic cells, macrophages, and colloid-like material in smears leads to misinterpretation of a PA as a thyroid lesion. Changes in the Hürthle cells in thyroidal follicular cells appear similar to changes in oxyphil cells in PA.^4^ It is especially difficult to differentiate a parathyroid lesion from a thyroid neoplasm when microfollicular features are present. In our index case, the presence of clusters and pseudoacini which closely resemble the microfollicles of follicular thyroid neoplasm, and the absence of a colloid in the background prompted us to consider the possibility of follicular neoplasm (Bethesda category IV) on FNA. However, there are subtle clues that could differentiate PA from thyroid lesions. The nuclei of PA are usually small and show more hyperchromasia. Stippled chromatin is noted in a subset of cases. The nuclei of thyroid tumors are usually more open. Intranuclear inclusion, although considered characteristic of papillary thyroid carcinoma, is rarely noted in PA.^15^ Cell block preparation. Further, IHC on the FNA material can be performed to distinguish parathyroid and thyroid neoplasm. Also, evaluation of the PTH level in FNA washout material is also helpful.^15^ However, cytology cannot distinguish the types of parathyroid neoplasm. Capsular, vascular, lymphatic, and perineural invasion, essential for diagnosing carcinoma, cannot be identified in FNA smears. Hence, surgical excision is the only diagnostic method to diagnose APT or parathyroid carcinoma.

USG is highly sensitive, inexpensive, and non-radioactive, but it is operator-dependent and requires skill and experience to pick up abnormal parathyroid lesions.^17^ Large parathyroid neoplasms, particularly those cases which are intrathyroidal in location, can mimic thyroid nodules. In our index case, the initial USG interpretation was that of a suspicious thyroid neoplasm (TI-RADS 5). Recent advancements in nuclear medicine imaging can reliably differentiate parathyroid tumors from thyroid neoplasms. Tc-99m MIBI SPECT/CT has been considered the initial diagnostic choice, as it shows cervical and mediastinal lesions with a high positive predictive value of more than 80%.^17^ Although the reported sensitivity of Tc-99m MIBI SPECT/CT is high, it may not be sufficient for preoperative localization of parathyroid adenomas when used alone.^18^ Another important confounding factor affecting the diagnostic value of Tc-99m MIBI SPECT/CT is concomitant thyroid nodules, which are known to be more frequent in primary hyperparathyroidism patients.^19^ Some thyroid nodules exhibit intense MIBI uptake early with no tendency to washout or delayed washout, thereby mimicking parathyroid adenomas.^17^Hence, any diagnostic modality alone is not gold-standard to distinguish parathyroid adenoma and thyroid neoplasm, and a clinical, biochemical, imaging, and pathological correlation is required. Concomitant thyroid and parathyroid malignancy, although very rare, has been reported in the literature.^20^

The management protocol of APT is not well documented because of the rarity of the lesion. Excision of tumors with free surgical margins is the mainstay of treatment. The recurrence rate is variable, ranging from 0 to 3.7% in most of the literature. No consensus has been made regarding adjuvant chemotherapy or radiotherapy in APT.^6^

## CONCLUSION

This case is an example where a rare neoplasm of the parathyroid gland can cause immense diagnostic challenges among clinicians, radiologists, and pathologists. A combined clinical radiological and pathological approach is necessary. Fine needle aspiration in parathyroid tumors has limited utility. The diagnosis of APT can only be established on extensive histological evaluation.

## References

[1] Gokozan HN, Scognamiglio T (2023). Advances and updates in parathyroid pathology. Adv Anat Pathol.

[2] Erickson LA, Mete O, Juhlin CC, Perren A, Gill AJ (2022). Overview of the 2022 WHO classification of parathyroid tumors. Endocr Pathol.

[3] Christakis I, Busaidy NL, Cote GJ (2016). Parathyroid carcinoma and atypical parathyroid neoplasms in MEN1 patients: a clinico-pathologic challenge. The MD Anderson case series and review of the literature. Int J Surg.

[4] Sriphrapradang C, Sornmayura P, Chanplakorn N, Trachoo O, Sae-Chew P, Aroonroch R (2014). Fine-needle aspiration cytology of parathyroid carcinoma mimic hürthle cell thyroid neoplasm. Case Rep Endocrinol.

[5] Agarwal A, Fernando R, Parameswaran R, Mishra A, Pradhan R (2023). Case studies in thyroid and parathyroid tumors..

[6] Cetani F, Marcocci C, Torregrossa L, Pardi E (2019). Atypical parathyroid adenomas: challenging lesions in the differential diagnosis of endocrine tumors. Endocr Relat Cancer.

[7] McCoy KL, Seethala RR, Armstrong MJ (2015). The clinical importance of parathyroid atypia: is long-term surveillance necessary?. Surgery.

[8] Alwaheeb S, Rambaldini G, Boerner S, Coiré C, Fiser J, Asa SL (2006). Worrisome histologic alterations following fine-needle aspiration of the parathyroid. J Clin Pathol.

[9] Terroir M, Grimaldi S, Hartl D, Leboulleux S, Deandreis D (2019). Parathyroid glands hyperplasias mimicking medullary thyroid carcinoma metastatic lymph nodes on 18F-DOPA PET/CT. Clin Nucl Med.

[10] Ordóñez NG (2014). Value of GATA3 immunostaining in the diagnosis of parathyroid tumors. Appl Immunohistochem Mol Morphol.

[11] Truran PP, Johnson SJ, Bliss RD, Lennard TW, Aspinall SR (2014). Parafibromin, galectin-3, PGP9.5, Ki67, and cyclin D1: using an immunohistochemical panel to aid in the diagnosis of parathyroid cancer. World J Surg.

[12] Silva-Figueroa AM, Bassett R, Christakis I (2019). Using a novel diagnostic nomogram to differentiate malignant from benign parathyroid neoplasms. Endocr Pathol.

[13] Uljanovs R, Sinkarevs S, Strumfs B, Vidusa L, Merkurjeva K, Strumfa I (2022). Immunohistochemical profile of parathyroid tumours: a comprehensive review. Int J Mol Sci.

[14] Chen Y, Song A, Nie M (2023). Clinical and genetic analysis of atypical parathyroid adenoma compared with parathyroid carcinoma and benign lesions in a Chinese cohort. Front Endocrinol.

[15] Ha HJ, Kim EJ, Kim JS (2020). Major clues and pitfalls in the differential diagnosis of parathyroid and thyroid lesions using fine needle aspiration cytology. Medicina.

[16] Heo I, Park S, Jung CW (2013). Fine needle aspiration cytology of parathyroid lesions. Korean J Pathol.

[17] Lu R, Zhao W, Yin L (2021). Efficacy of ultrasonography and Tc-99m MIBI SPECT/CT in preoperative localization of parathyroid adenomas causing primary hyperthyroidism. BMC Med Imaging.

[18] Zeng M, Liu W, Zha X (2019). ^99m^Tc-MIBI SPECT/CT imaging had high sensitivity in accurate localization of parathyroids before parathyroidectomy for patients with secondary hyperparathyroidism. Ren Fail.

[19] Li L, Li B, Lv B (2021). Increased thyroid malignancy in patients with primary hyperparathyroidism. Endocr Connect.

[20] Cuhaci N, Ozdemir D, Polat B (2017). Concomitant thyroid lesions in patients with primary hyperparathyroidism. Asian J Surg.

